# High spatial resolution (10–50 μm) analysis of Sr isotopes in rock-forming apatite by LA-MC-ICP-MS†

**DOI:** 10.1039/d3ja00177f

**Published:** 2023-08-29

**Authors:** Anda Buzenchi, Hugo Moreira, Olivier Bruguier, Delphine Bosch, Bruno Dhuime

**Affiliations:** a Géosciences Montpellier, CNRS & Université de Montpellier Bât 22 CC60, Place Eugene Bataillon 34095 Montpellier Cedex 5 France anda-ioana.buzenchi@umontpellier.fr

## Abstract

*In situ* Sr isotopes analysis of apatite by LA-(MC)-ICP-MS is challenged by the difficulty to monitor and correct isobaric interferences from atomic and polyatomic ions. We present a new routine procedure for analysing rock-forming apatites with a Thermo Scientific Neptune XT MC-ICP-MS coupled with a Teledyne Cetac Analyte Excite+ 193 nm laser ablation system. Five apatite standards that cover a large range of REE/Sr ratios were selected, and their ^87^Sr/^86^Sr ratios were measured in solution after dissolution and purification of Sr [Durango: 0.706321(5); Madagascar: 0.711814(5); Slyudyanka; 0.707705(4); Sumé: 0.707247(4); and Ipirá: 0.710487(4)]. The optimisation of both instrument setup and data reduction schemes was achieved through repeated measurements of calibration solutions and of apatite standards at four different rectangular-shaped laser ablation beam sizes (50 × 50, 25 × 25, 13 × 13 and 10 × 10 μm). Two complementary methods were developed for data reduction: *Method 1*, which corrects measured intensities for gas blank and instrumental mass bias only; and *Method 2*, which additionally corrects for isobaric interferences of ^87^Rb^+^, ^166, 168 and 170^Er^++^, ^170, 172, 174 and 176^Yb^++^, ^40^Ca^44^Ca^+^, ^40^Ca^46^Ca^+^, ^44^Ca^43^Ca^+^ and ^40^Ca^48^Ca^+^. A precision of *ca.* 100 ppm (2 s.e.) can be achieved on the ^87^Sr/^86^Sr ratio with a 50 μm laser ablation beam when using *Method 2*, and it remains better than 3000 ppm at 10 μm with *Method 1*. *Method 1* gives precise and accurate ^87^Sr/^86^Sr ratios when ^173^Yb^++^ is below the global limit of detection (with LOD_global_ = 3 s.d. of the means of all gas blanks measurements). When ^173^Yb^++^ is above the LOD_global_, *Method 2* should be preferred as it provides more accurate ^87^Sr/^86^Sr ratios. Overall, this study offers a robust and reliable approach for LA-MC-ICP-MS analysis of Sr isotopes in rock-forming apatite at a high spatial resolution (*i.e.* down to 10 μm), overcoming previous limitations associated with instrumental set up and data reduction.

## Abstract 2

This study presents a new routine procedure for LA-MC-ICPMS analyses of Sr isotopes in apatite with ablation spot sizes down to 10 × 10 μm.

## Graphical abstract

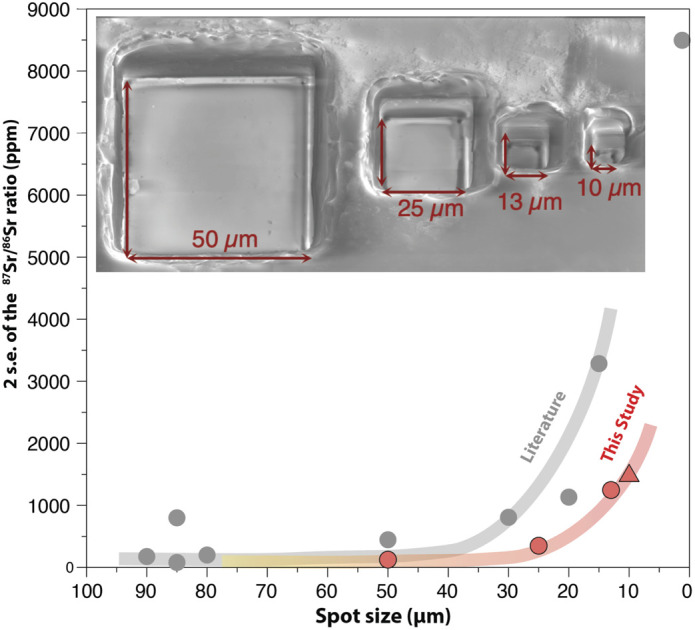

## 1. Introduction

Strontium isotopes have applications in many fields of research, including Earth and planetary sciences,^1,2^ archaeology,^3,4^ palaeontology,^5,6^ environmental sciences,^7–13^ forensic sciences^14,15^ and agriculture.^16–20^ In Earth and planetary sciences, the Rb–Sr system is commonly used to constrain the age and the magmatic source of bulk rocks^21–23^ and Sr-rich minerals such as apatite,^24–29^ plagioclase,^30–33^ phlogopite,^34^ or maskelynites from Martian meteorites.^35^ If the geological material has not experienced secondary processes of metamorphism and/or alteration with time, the ^87^Sr/^86^Sr ratio can be used to constrain the petrogenesis of igneous rocks,^36–40^ to unravel magma sources in young volcanoes and magmatic provinces,^41–46^ to date igneous rocks^22,23^ and to monitor the formation and the evolution of Earth's geochemical reservoirs through time.^47–50^

Strontium isotopes were initially analysed by thermal ionization mass spectrometry (TIMS), after separation and purification of Sr by ion exchange chromatography.^51–53^ In recent years, *in situ* analyses of Sr isotopes have advanced significantly, mirroring technical improvements in mass spectrometry. Both SIMS and LA-MC-ICP-MS techniques have been developed in parallel,^29,54,55^ and if one important challenge with SIMS is to find standards with Sr and rare earth elements (REE) concentrations matching those of the samples analysed, the major difficulty with LA-MC-ICP-MS is related to the presence of isobaric interferences from both atomic and polyatomic ions. As a consequence, Sr isotopes have remained challenging to analyse with a high level of precision and accuracy by LA-MC-ICP-MS. For instance, the interference of ^87^Rb^+^ on ^87^Sr^+^ induces a large uncertainty in the ^87^Sr/^86^Sr measured in samples with high Rb/Sr ratios, and accurate ^87^Sr/^86^Sr can hardly be obtained in samples with Rb/Sr > 0.3.^28,56–61^

Amongst low Rb/Sr (<0.01) minerals, apatite ((Ca_5_PO_4_)_3_(OH, Cl, F)) has received increasing interest over the last two decades. This accessory phase is commonly found in magmatic rocks and sediments, it can be dated with the U–Th–Pb method,^62–64^ and its ^87^Sr/^86^Sr ratio remains unchanged since its crystallisation due to the negligible ingrowth of ^87^Sr from the radiogenic decay of ^87^Rb. As a consequence, apatite has become a mineral of choice besides zircon in crustal evolution studies, although dissolution and recrystallisation under fluid circulation can drastically alter the ^87^Sr/^86^Sr ratio of apatites present in the rock matrix.^65,66^ To overcome this issue, recent studies have focused on measuring Sr isotopes in minute (*i.e.* 10–50 μm) apatite inclusions in more refractory and resistant minerals such as zircon.^24,67–69^

Apatites have relatively high concentrations of calcium, phosphorus, erbium and ytterbium, which produce a number of isobaric interferences on ^84^Sr^+^, ^86^Sr^+^, ^87^Sr^+^ and ^88^Sr^+^.^56,70^ Previous studies have presented different protocols for doubly-charged REE interference corrections, and the most common approach is to monitor interference-free doubly-charged ions on half-masses (*e.g.*, ^167^Er^++^ on *m*/*z* 83.5; ^173^Yb^++^ on *m*/*z* 86.5) and to use natural isotopic abundances to calculate the contribution of interfering species.^56,70^ The isobaric interference of ^40^Ca^31^P^16^O^+^ (or CaPO^+^) on ^87^Sr^+^ can be drastically reduced by controlling the instrumental conditions to reduce oxide production in the plasma,^71,72^ although this generally results an overall decrease of sensitivity that affects analytical precision. Finally, although calcium dimers and argides interferences on Sr isotopes have been reported,^26,73,74^ their influence on the ^87^Sr/^86^Sr ratio remains debated and is often considered insignificant.^56,57,61^

In this study we present a combination of new instrumental conditions and data reduction protocols for LA-MC-ICP-MS, and we apply this approach to the analysis of five apatite reference materials (Durango;^75^ Madagascar;^76^ Slyudyanka;^63^ Sumé;^77^ Ipirá), using laser ablation beam sizes between 50 × 50 μm and 10 × 10 μm. Both the precision and the accuracy of the ^87^Sr/^86^Sr ratio are improved compared to previous studies, which opens new perspectives for Sr isotope analyses at high spatial resolution.

## 2. Materials and methods

### 2.1. Rock-forming apatites used as reference material

Five apatites with a large range of Sr and REE contents were selected for this study. Madagascar, Durango, Slyudyanka and Sumé apatites were characterised in previous studies (Table 1), and Ipirá is a new in-house standard (C. Lana, *Pers. Comm.*). To ensure that the Sr isotope composition of the apatite fragments selected for LA-MC-ICP-MS analyses is consistent with the literature data, 40 to 100 mg of these fragments were dissolved in tri-distilled HNO_3_ 65% at 140 °C, and Sr was isolated in Teflon columns filled with Eichrom Sr-Spec ion-exchange resin following the approach of Pin *et al.*^78^ Sr solutions were analysed with a Thermo Scientific MC-ICP-MS Neptune Plus at the AETE-ISO Platform (OSU-OREME, University of Montpellier), and the new Sr isotope data are summarised in Table 2.

**Table 1:** Summary of published data for the apatites used as reference material in this study

	Sr (ppm)	Er (ppm)	Yb (ppm)	Rb (ppm)	Er + Yb/Sr	^87^Sr/^86^Sr	2 s.d.	*n*	Method	Reference
Durango						0.706330	0.000030	3	TIMS^a^	Horstwood *et al.*^26^
	486	34–83	27–59	0.13	0.12 to 0.28	0.706340	0.000140	156	LA-MC-ICPMS	Yang *et al.*^29^
						0.706328	0.000023	13	TIMS + MC-ICPMS^a^	Yang *et al.*^29^
						0.706346	0.000516		LA-MC-ICPMS	Cao *et al.*^30^
Madagascar	1650	21	15	0.25	0.021	0.711800	0.000010	112	LA-MC-ICPMS	Yang *et al.*^29^
						0.711800	0.000002	11	TIMS + MC-ICPMS^a^	Yang *et al.*^29^
						0.712300	0.003000	6	CHILI	Boehnke *et al.*^67^
						0.712300	0.001800	43	SIMS	Gillespie *et al.*^25^
						0.711879	0.000157		LA-MC-ICPMS	Cao *et al.*^30^
						0.711900	0.000100	10	LA-MC-ICPMS	Huang *et al.*^79^
Slyudyanka	1231	2	4	0.02	∼0.005	0.707690	0.000015	110	LA-MC-ICPMS	Yang *et al.*^29^
						0.707680	0.000003	11	TIMS + MC-ICPMS^a^	Yang *et al.*^29^
						0.708220	0.001690	28	SIMS	Gillespie *et al.*^25^
Sumé	730–806	20–23	13–15	<0.1	∼0.045	0.708000	0.000200	14	LA-MC-ICPMS	Lana *et al.*^77^
Ipirá	256	17–7	12–6	<1.6	0.05 to 0.11	0.71049	0.00028	33	LA-MC-ICPMS	Cristiano Lana, *personal communication*

a Analysis after chemical purification using conventional chromatographic column separation.

**Table 2:** Summary of solution MC-ICP-MS analyses of the five apatite standards selected for LA-MC-ICP-MS analyses

Sample name	^88^Sr(v)	^87^Sr/^86^Sr	2 s.e.
Durango_1	8.7	0.706319	0.000005
Durango_2	6.6	0.706322	0.000005
Madagascar	11.4	0.711814	0.000003
Slyudyanka_1	16.5	0.707705	0.000003
Slyudyanka_2	14.4	0.707706	0.000004
Sumé_1	10.5	0.707250	0.000003
Sumé_2	9.7	0.707245	0.000004
Ipirá	7.6	0.710487	0.000004

#### 2.1.1. Durango

Durango is a widely used reference material, which is sampled in an open pit iron mine near the Durango City in Mexico. It is a yellow-green fluorapatite, with a ID-TIMS U–Pb age of 32.716 ± 0.061 Ma.^75^ It has the lowest Sr concentration (486 ppm) and the highest REE contents (Er = 34–83 ppm and Yb = 27–59 ppm) and (Er + Yb)/Sr ratio amongst apatites selected for this study (Table 1). The ^87^Sr/^86^Sr ratio of 0.706328 ± 0.000023 that Yang *et al.* obtained by TIMS and solution MC-ICP-MS was chosen as reference value.^29^ Our new data for two fragments of Durango overlap within error (Table 2), with a mean ^87^Sr/^86^Sr = 0.706321 ± 0.000005 within error of the Yang *et al.* value.^29^

#### 2.1.2. Madagascar

Madagascar (or MAD) is a large fragment of apatite from the First Mine Discovery in Madagascar. Due to its homogenous U–Pb isotope composition, it has been widely used as a reference material for U–Pb dating, with a reference age of 474.25 ± 0.41 Ma.^76^ It has a very high Sr concentration (1650 ppm), relatively high REE contents (Er = 21 ppm and Yb = 15 ppm), and its (Er + Yb)/Sr ratio is about 10 times greater than in Durango apatite (Table 1). The ^87^Sr/^86^Sr ratio of 0.711800 ± 0.000026 that Yang *et al.*^29^ obtained by TIMS and solution MC-ICP-MS was chosen as reference value. The ^87^Sr/^86^Sr of 0.711814 ± 0.000005 that we obtained for one fragment of this apatite (Table 2) is within error of the Yang *et al.* value (Table 1).^29^

#### 2.1.3. Slyudyanka

Slyudyanka originates from the Slyudyanka complex, which is located on the southwest coast of Lake Baikal in Russia. It has a U–Pb age of 447.0 ± 7.3 Ma,^63^ a high Sr concentration (1231 ppm), very low REE contents (Er = 5 ppm and Yb = 4 ppm) and the lowest (Er + Yb)/Sr ratio amongst apatites selected for this study (*i.e.* about 40 times lower than in Durango apatite; Table 1). The ^87^Sr/^86^Sr ratio of 0.707683 ± 0.000025 that Yang *et al.*^29^ obtained by TIMS and solution MC-ICP-MS was chosen as reference. Our new data for two fragments of Slyudyanka overlap within error (Table 2), with a mean ^87^Sr/^86^Sr = 0.707706 ± 0.000004 within error of the Yang *et al.* value (Table 1).^29^

#### 2.1.4. Sumé

Sumé was recently proposed as a reference material by Lana *et al.*^77^ It comes from the Borborema Province in NE Brazil and is found as large pockets of green-blueish apatite mineralisation within felsic gneisses of the Foresta Complex. It has a ID-TIMS U–Pb age of 568 ± 2 Ma,^77^ a relatively high concentration of Sr (730–800 ppm) and REE (Yb = 20–23 ppm and Er = 13–15 ppm), and its (Er + Yb)/Sr ratio is about 4–5 times greater than in Durango apatite (Table 1). Lana *et al.* obtained an average ^87^Sr/^86^Sr isotope ratio of 0.7080 ± 0.0002 by LA-MC-ICP-MS, although they reported Sr isotope heterogeneities within different grains, with ^87^Sr/^86^Sr ranging between 0.70795 ± 0.00017 and 0.70850 ± 0.00026.^77^ Our new data for the two fragments of Sumé overlap within error (Table 2), with a mean ^87^Sr/^86^Sr = 0.707247 ± 0.000004 that is significantly lower than the mean value of Lana *et al.* (0.7080 ± 0.0002).^77^

#### 2.1.5. Ipirá

Ipirá is a colourless to green-blue apatite from the *ca.* 2 Ga Ipirá pegmatite in the State of Bahia in Brazil.^80^ It frequently contains micrometric solid inclusions of unknown composition, and to our knowledge no data have been published for this apatite. The ^87^Sr/^86^Sr of 0.710487 ± 0.000004 that we obtained for one fragment of Ipirá (Table 2) is consistent with preliminary LA-MC-ICP-MS data from the Universidade Federal de Ouro Preto in Brazil (C. Lana, *personal communication*).

### 2.2. Analytical conditions

Analyses were performed at the MILESTONE laboratory (Géosciences Montpellier), which is part of the ISOTOP-MTP platform of the RéGEF (https://www.regef.fr/). We used a Thermo Scientific Neptune XT multicollector inductively coupled plasma mass spectrometer (MC-ICP-MS), for which the sensitivity was improved by using an Edwards nXL110i dry interface pump in combination with a Jet sample cone and a X skimmer cone. Typical operating conditions are outlined in Table 3 and the Faraday cup configuration is detailed in Table 4.

**Table 3:** Typical operating conditions for (LA)-MC-ICP-MS analyses

	Laser ablation	Solution
**Neptune XT MC-ICP-MS**		
RF power (W)	1350	1200
Focus voltage	−609	−611
Cool gas (L min^−1^)	15	15
Auxiliary gas (L min^−1^)	0.8	0.8
Sample gas (L min^−1^)	1.026	1.100

**Analyte Excite + with HelEx II 2-volumes ablation cell**		
MFC1 He (L min^−1^)	0.35	
MFC2 He (L min^−1^)	0.17	
MFC3 N_2_ (mL min^−1^)	10	
Repetition rate (Hz)	5	
Fluence (J cm^−2^)	6	

**Table 4:** Neptune cup configuration for Sr isotope analyses, and potential isobaric interferences for each mass

Faraday cup Amplifier (ohm)	L4 10^11^	L3 10^11^	L2 10^11^	L1 10^11^	C 10^11^	H1 10^11^	H2 10^11^	H3 10^11^	H4 10^11^
*m*/*z*	83	83.5	84	85	85.5	86	86.5	87	88
Sr			^84^Sr^+^			^86^Sr^+^		^87^Sr^+^	^88^Sr^+^
Rb				^85^Rb^+^				^87^Rb^+^	
Kr	^83^Kr^+^		^84^Kr^+^			^86^Kr^+^			
Er	^166^Er^++^	^167^Er^++^	^168^Er^++^	^170^Er^++^					
Yb			^168^Yb^++^	^170^Yb^++^	^171^Yb^++^	^172^Yb^++^	^173^Yb^++^	^174^Yb^++^	^176^Yb^++^
Lu									^176^Lu^++^
Hf									^176^Hf^++^
Ca dimers	^40^Ca^43^Ca^+^		^42^Ca^42^Ca^+^			^42^Ca^44^Ca^+^		^43^Ca^44^Ca^+^	^40^Ca^48^Ca^+^
			^40^Ca^44^Ca^+^			^40^Ca^46^Ca^+^			^44^Ca^44^Ca^+^
						^43^Ca^43^Ca^+^			^42^Ca^46^Ca^+^
Ca argides	^43^Ca^40^Ar^+^		^44^Ca^40^Ar^+^			^46^Ca^40^Ar^+^			^48^Ca^40^Ar^+^
			^46^Ca^38^Ar^+^			^48^Ca^38^Ar^+^			
			^48^Ca^36^Ar^+^						

For *in situ* analyses, the MC-ICP-MS was coupled to a Teledyne Cetac Analyte Excite + excimer laser, which includes an optional ‘*X*–*Y* Theta’ dynamic aperture that allows rectangular-shaped laser beams of any aspect ratio and orientation to be generated. Tuning for optimal sensitivity, signal stability and best peak shape were performed daily in rastering ablation mode on the Durango apatite, with a 50 × 50 μm laser ablation beam, a scan speed of 5 μm s^−1^, a fluence of 6 J cm^−2^ and a repetition rate of 6 Hz. Oxide production in the plasma was monitored with the UO^+^/U^+^ ratio, and optimal plasma conditions (UO^+^/U^+^ < 0.1%) were achieved by tuning the torch position and the Ar, He and N_2_ gas flow rates. Apatite analyses were done at four different laser ablation beam sizes: 50 × 50 μm, 25 × 25 μm, 13 × 13 μm and 10×10 μm. The integration time of the Neptune was set at 1 s per cycle. A gas line wash-out of 40 cycles and an on-peak gas blank of 120 cycles were analysed before each apatite measurement of 60 cycles.

The way particles are transported from the ablation cell towards the plasma is key to achieve high-precision measurements by LA-MC-ICP-MS.^81,82^ Several configurations have been tested, including the smoothing “SQUID” manifold that splits the mixture of gas and sample aerosol flow into ten nylon tubes of differing lengths, such that after recombination into one gas line a smooth signal is produced;^81^ and the Aerosol Rapid Introduction System (ARIS) that consists of a 1 m long polyether ether-ketone (PEEK) capillary tubing that penetrates the injector with a specific adapter to the torch. The ARIS allows a more efficient transfer of the particles from the ablation site to the torch, while also decreasing oxide formation.^82^ For Sr isotopes, a 2–3 times improvement of both the instrumental sensitivity and the internal precision of the measurements was observed with the ARIS (ESI Fig. 1†), which is why this system was selected for this study.

For solution analyses, solutions were sprayed to the instrument's torch *via* a 100 μL min^−1^ nebuliser and a cyclonic spray chamber. The instrumental sensitivity was 0.3 V ppb^−1^ for Sr isotopes, and 40 cycles of 1 s each were integrated for each measurement.

**Fig. 1:**
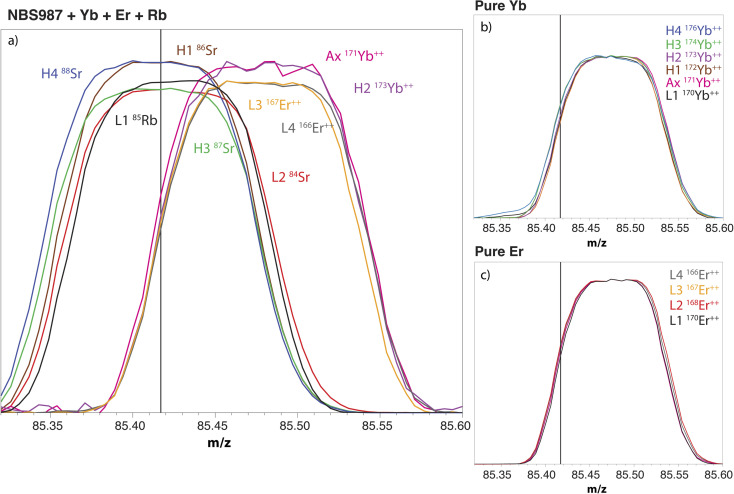
Mass scans of (a) a solution of 100 ppb NIST SRM 987 doped with 20 ppb Yb, 20 ppb Er and 5 ppb Rb; (b) a solution of pure Yb; and (c) a solution of pure Er. Factors were applied to the intensities measured in order to obtain peaks with similar heights. The vertical line indicates the *m*/*z* set for the central cup.

The MC-ICP-MS cup configuration that is commonly used to measure Sr isotopes involves the alignment of the cups measuring interference-free doubly-charged Er^++^ and Yb^++^ ions (*i.e. m*/*z* = 83.5 for ^167^Er^++^, 85.5 for ^171^Yb^++^ and 86.5 for ^173^Yb^++^) with the other cups measuring Sr and Rb isotopes. The on-peak measurements of ^171^Yb^++^, ^173^Yb^++^ and ^167^Er^++^, and the natural ratios between these isotopes and the Yb^++^ and Er^++^ ions interfering on *m*/*z* 84, 85, 86, 87 and 88, are commonly used to calculate the intensities of interference-free Rb and Sr isotopes.^56,70^ However, and as a major limitation of this approach, doubly-charged Er and Yb ions have a peak high that is shifted by *ca.* 0.05–0.07 amu from the peak highs of the Rb and Sr ions on which they interfere (Fig. 1).^56,70^ As a result, the on-peak measurement of ^171^Yb^++^, ^173^Yb^++^ and ^167^Er^++^ leads to an overestimation of the intensities of the interfering Yb^++^ and Er^++^ ions on the cups measuring Rb and Sr isotopes. To avoid this issue, we used a new approach in which the interference-free doubly-charged ^171^Yb^++^, ^173^Yb^++^ and ^168^Er^++^ were aligned with the Yb^++^ and Er^++^ ions interfering on *m*/*z* 84, 85, 86, 87 and 88 (Fig. 1).

### 2.3. Interferences and mass bias correction

The raw data intensities were reduced offline using an in-house Excel spreadsheet. For each *m*/*z* of interest, the mean intensities of the gas blank were calculated after applying a 2 s.d. threshold for outlier rejection, and the gas blank was subtracted from the raw intensities measured in the sample. We assumed that krypton is only present in the gas and that the contribution of Kr on *m*/*z* 83, 84 and 86 is negligible after gas blank correction. Our approach for the correction of interferences on *m*/*z* 84, 85, 86, 87 and 88 includes three successive steps, after which a fourth step of mass bias correction was applied.

#### 2.3.1. Step 1: Doubly-charged Er and Yb correction

Interferences produced by doubly-charged Er and Yb ions can significantly alter the measurement of Sr isotopes by LA-MC-ICP-MS. ^166^Er^++^, ^168^Er^++^ and ^170^Er^++^ interfere on masses 83, 84 and 85, respectively, while ^170^Yb^++^, ^172^Yb^++^, ^174^Yb^++^ and ^176^Yb^++^ interfere on masses 85, 86, 87 and 88, respectively^56,61,70^ (Table 2).

For ytterbium, the interference-free doubly-charged ^173^Yb^++^ was measured on *m*/*z* 86.5. The ratios used to calculate the amount of doubly-charged Yb ions interfering on each mass of interest were defined from the analysis pure Yb solutions, with ^168^Yb/^173^Yb = 0.007651; ^170^Yb/^173^Yb = 0.185183; ^172^Yb/^173^Yb = 1.163427; ^174^Yb/^173^Yb = 2.250953; and ^176^Yb/^173^Yb = 0.795370. Yb-free intensities were calculated from eqn (1)–(5), in which the subscripts *m* and *IC* are for *measured* and *interference-corrected* intensities, respectively:

1 ^84^Sr^+^_YbIC_ = ^84^Sr^+^_m_ – ^168^Yb/^173^Yb_(0.007651)_ × ^173^Yb^++^_m_

2 ^85^Rb^+^_YbIC_ = ^85^Rb^+^_m_ – ^170^Yb/^173^Yb_(0.185183)_ × ^173^Yb^++^_m_

3 ^86^Sr^+^_YbIC_ = ^86^Sr^+^_m_ – ^172^Yb/^173^Yb_(1.163427)_ × ^173^Yb^++^_m_

4 ^87^Sr^+^_YbIC_ = ^87^Sr^+^_m_ – ^174^Yb/^173^Yb_(2.250953)_ × ^173^Yb^++^_m_

5 ^88^Sr^+^_YbIC_ = ^88^Sr^+^_m_ – ^176^Yb/^173^Yb_(0.795370)_ × ^173^Yb^++^_m_

For erbium, the interference-free doubly-charged ^167^Er^++^ was measured on *m*/*z* 83.5. The ratios used to calculate the amount of doubly-charged Er ions interfering on each mass of interest were defined from the analysis of pure Er solutions, with ^170^Er/^167^Er = 0.640024; ^168^Er/^167^Er = 1.275114; and ^166^Er/^167^Er = 1.536252. Er-free intensities were calculated from eqn (6)–(8), in which the subscripts *m* and *IC* are for *measured* and *interference-corrected* intensities, respectively:

6 ^40^Ca^43^Ca^+^_ErIC_ = ^40^Ca^43^Ca^+^_m_ – ^166^Er/^167^Er_(1.536252)_ × ^167^Er^++^_m_

7 ^84^Sr^+^_ErIC_ = ^84^Sr^+^_YbIC_ – ^168^Er/^167^Er_(1.275114)_ × ^167^Er^++^_m_

8 ^85^Rb^+^_ErIC_ = ^85^Rb^+^_YbIC_ – ^170^Er/^167^Er_(0.640024)_ × ^167^Er^++^_m_

#### 2.3.2. Step 2: Ca dimers correction

We assumed that the remaining intensity on *m*/*z* 83 after subtracting ^83^Kr^+^ and ^166^Er^++^ is entirely due to the interference of ^40^Ca^43^Ca^+^. The ratios used to calculate the amount of Ca dimers interfering on each mass of interest were the natural ratios published by Rosman and Taylor (^40^Ca^44^Ca/^40^Ca^43^Ca = 15.451850; ^40^Ca^46^Ca/^40^Ca^43^Ca = 0.029630; ^44^Ca^43^Ca/^40^Ca^43^Ca = 0.021518; ^40^Ca^48^Ca/^40^Ca^43^Ca = 1.385185).^83^ Ca dimers-free intensities were calculated from eqn (9)–(12), in which the subscripts *IC* are for *interference-corrected* intensities:

9 ^84^Sr^+^_CaIC_ = ^84^Sr^+^_ErIC_ – ^40^Ca^44^Ca/^40^Ca^43^Ca_(15.451850)_ × ^40^Ca^43^Ca^+^_ErIC_

10 ^86^Sr^+^_CaIC_ = ^86^Sr^+^_ErIC_ – ^40^Ca^46^Ca/^40^Ca^43^Ca_(0.029630)_ × ^40^Ca^43^Ca^+^_ErIC_

11 ^87^Sr^+^_CaIC_ = ^87^Sr^+^_ErIC_ – ^44^Ca^43^Ca/^40^Ca^43^Ca_(0.021518)_ × ^40^Ca^43^Ca^+^_ErIC_

12 ^88^Sr^+^_CaIC_ = ^88^Sr^+^_ErIC_ – ^40^Ca^48^Ca/^40^Ca^43^Ca_(1.385185)_ × ^40^Ca^43^Ca^+^_ErIC_

We note however that the Ca dimers correction produces insignificant changes in the calculation of the ^87^Sr/^86^Sr ratio at the level of precision achieved in our study. This is consistent with the previous observations of Ramos *et al.*, Yang *et al.*, and Irrgeher *et al.*^56,57,70^

#### 2.3.3. Step 3: Rb correction

The interference-free ^85^Rb^+^ was measured on *m*/*z* 85, and the ratio used to calculate the amount of ^87^Rb^+^ interfering on ^87^Sr^+^ was defined from repeated measurements of NIST SRM 987 solutions spiked with pure Rb solutions (^87^Rb/^85^Rb = 2.589745), following the approach described by Horstwood *et al.*^26^ Since only one rubidium isotope is interference-free (*i.e.*^85^Rb^+^; Table 4), the mass bias of Rb (βRb) could not be calculated during apatite analyses and βSr was used instead of βRb, assuming that instrumental mass bias discrimination is reasonably similar for Rb and Sr.^26,27^ Rb-free intensities were calculated from eqn (13), in which the subscripts *m* and *IC* are for *measured* and *interference-corrected* intensities, respectively:

13 ^87^Sr^+^_RbIC_ = ^87^Sr^+^_CaIC_ – ^85^Rb^+^_ErIC_ × (^87^Rb/^85^Rb)_(2.589745)_/*e*^(βSr×ln(M87/M85))^

with:βSr = ln(^88^Sr/^86^Sr_natural_/^88^Sr/^86^Sr_m_)/ln(M88/M86)

#### 2.3.4. Step 4: Mass bias correction

Finally, an exponential mass bias correction was applied to calculate the final ^87^Sr/^86^Sr ratio, following eqn (14):

14 ^87^Sr/^86^Sr_mbc_ = ^87^Sr^+^_RbIC_/^86^Sr^+^_CaIC_ × (M87/M86)^βSr^

where ^87^Sr/^86^Sr_mbc_ is the final mass bias-corrected ratio, and (M87/M86)^βSr^ is the mass bias correction factor.

### 2.4. Validation of the approach used for data reduction

**Fig. 2:**
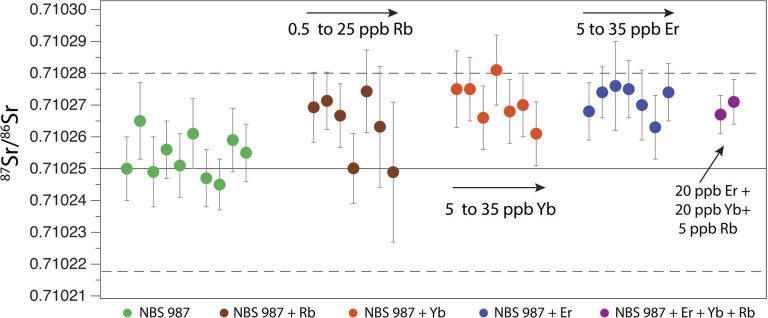
Interferences and mass bias corrected ^87^Sr/^86^Sr ratios in solutions containing pure NIST SRM 987 spiked with various concentrations of interfering species (Rb, Yb and Er). Error bars are 2 s.e. Horizontal solid and dashed lines represent the average value and the 2 s.d., respectively, of 1279 TIMS analyses of NIST SRM 987 reference material (downloaded from https://georem.mpch-mainz.gwdg.de/).

A summary of solution analyses of a pure Sr standard (NIST SRM 987), and of NIST SRM 987 spiked with various amounts of pure solutions of Rb, Er and Yb, is given in Fig. 2 and ESI Table 1.† For solutions containing interfering elements, analyses were separated into 5 groups of solutions that contain NIST SRM 987 as well as (1) Rb; (2) Yb; (3) Er; (4) Yb + Er; and (5) Rb + Yb + Er. When using the approach described in *Section 2.3* for interferences and mass bias correction, all ^87^Sr/^86^Sr ratios plot within error of the reference value of Thirlwall (*i.e.*^87^Sr/^86^Sr = 0.710248 ± 0.000024; 2 s.d.), and within error of high-precision TIMS values published for NIST SRM 987 in the past 20 years (0.710249 ± 0.000031). Overall, these results further validate our approach.

## 3. LA-MC-ICP-MS analyses

### 3.1. Impact of interferences on the ^87^Sr/^86^Sr ratio

**Fig. 3:**
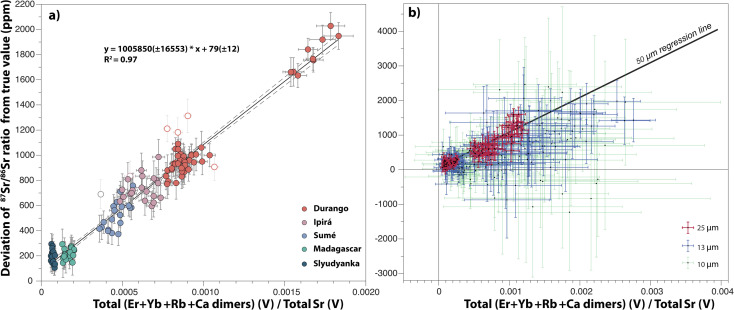
Accuracy of the mass bias corrected ^87^Sr/^86^Sr ratio (expressed as the deviation in ppm from the reference value) as a function of the contribution of interfering species on measured Sr intensities. (a) Apatite analyses with a 50 μm laser ablation beam size. Empty symbols were not used for the regression. (b) Apatite analyses at 25 μm, 13 μm and 10 μm.

A summary of 457 analyses performed on Durango, Madagascar, Slyudyanka, Sumé and Ipirá apatites at four laser ablation beam sizes (50 μm, 25 μm, 13 μm and 10 μm) is presented in ESI Table 2.† The deviation in ppm of the ^87^Sr/^86^Sr corrected only from mass bias, from the reference ^87^Sr/^86^Sr ratio of each apatite standard, is plotted as a function of the ratio of the sum of intensities of interfering species over the total Sr intensity in Fig. 3. A positive correlation is observed, and this correlation becomes more evident for analyses performed with the largest laser ablation beam size (*i.e.* 50 μm; Fig. 3a), as higher intensities are measured on Faraday cups. For smaller beam sizes, especially at 10 μm, the lower intensities measured on Faraday cups drastically alter the precision, and the need for correcting small interferences becomes less evident. This is particularly true for apatites with the lowest ratios of interfering species over total Sr (Fig. 3b).

**Fig. 4:**
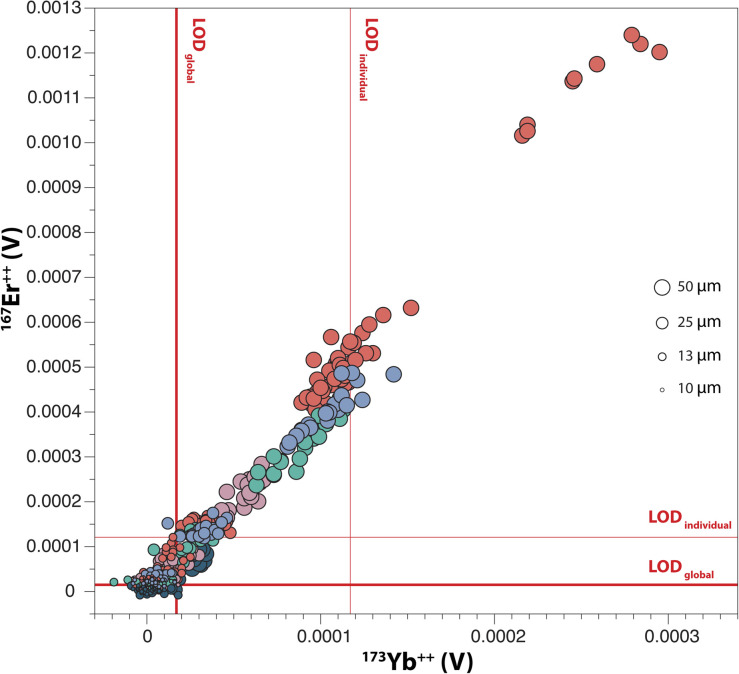
Variation of ^167^Er^++^ and ^173^Yb^++^ intensities measured in apatite standards, for laser ablation beam sizes ranging between 50 μm and 10 μm. Colour codes are same as in Fig. 3a. The LOD_individual_ (thin red lines) is the average 3 s.d. of individual gas blank intensities, and the LOD_global_ (thick red lines) is the 3 s.d. of 460 gas blank analyses.

^167^Er^++^ intensities measured on *m*/*z* 83.5 are plotted against ^173^Yb^++^ intensities measured on *m*/*z* 86.5 in Fig. 4. A positive correlation is observed, which is consistent with the similar behaviour of heavy REE in rock-forming apatites during crystallisation. The need for interferences correction is highly dependent on the extent to which small intensities can be detected. Accordingly, the limit of detection for intensities measured on *m*/*z* 83.5 and 86.5 was calculated in two different ways in Fig. 4: (i) as the 3 s.d. of individual gas blank measurements before each apatite analysis (LOD_individual_); and (ii) as the 3 s.d. of the mean values of all gas blanks (LOD_global_). The LOD_global_ and LOD_individual_ are taken here as the lower and upper bounds, respectively, of the threshold from which intensities measured on Faraday cups should be used for interferences correction. Based on this observation and the correlation evidenced in Fig. 3, we explored two different methods for data reduction of the five apatite standards:

• *Method 1*, in which the blank-corrected ^87^Sr/^86^Sr ratio is only corrected from the mass bias.

• *Method 2*, in which the blank-corrected ^87^Sr/^86^Sr ratio is corrected from both interferences and mass bias, following the approach detailed in *Section 2.3*.

### 3.2. Durango

**Fig. 5:**
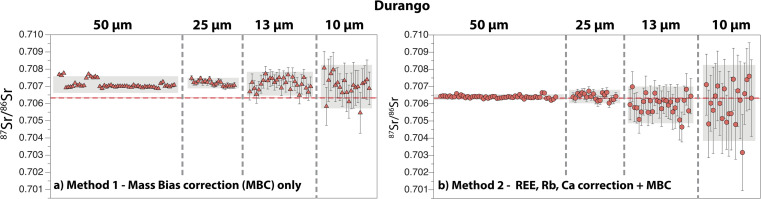
Compilation of ^87^Sr/^86^Sr ratios obtained by LA-ICP-MS on the Durango apatite, with laser ablation beam sizes ranging between 50 μm and 10 μm. (a) *Method 1* and (b) *Method 2* for data reduction (see text for details). Error bars are 2 s.e., with a number of symbols larger than error bars. Grey fields represent the 2 s.d. of repeated measurements at a given laser ablation beam size. Solid and dashed red lines are the published mean reference value and associated 2 s.d., respectively, for the Durango apatite.^29^

A summary of 121 Durango apatite analyses is presented in Fig. 5. The internal precision of individual measurements, which is given by the 2 s.e. of the mean of the number of cycles integrated, varies as a function of the laser ablation beam size and the method used. The 2 s.e. of the ^87^Sr/^86^Sr ratio ranges between 150 ppm (50 μm) and 1880 ppm (10 μm) for *Method 1*, and between 160 ppm (50 μm) and 3550 ppm (10 μm) for *Method 2*. Repeated measurements with *Method 2* give average ^87^Sr/^86^Sr ratios that are all within error of both the reference value and our new solution value (50 μm: 0.706392 ± 0.000163; 25 μm: 0.706421 ± 0.000391; 13 μm: 0.705935 ± 0.001069; 10 μm: 0.706047 ± 0.002208; 2 s.d.) (Tables 1 and 2; ESI Table 2†). When *Method 1* is used, a shift towards higher ^87^Sr/^86^Sr is observed, and only the average data at 10 μm are within error of the reference value (50 μm: 0.707106 ± 0.000484; 25 μm: 0.707190 ± 0.000305; 13 μm: 0.707153 ± 0.000692; 10 μm: 0.706985 ± 0.001266).

### 3.3. Madagascar

**Fig. 6:**
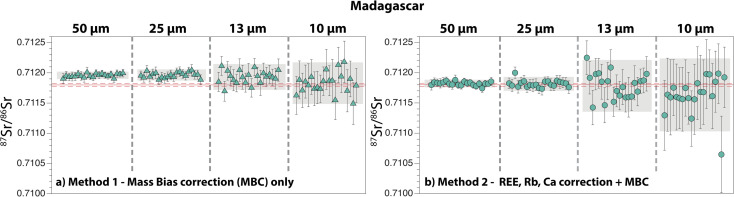
Compilation of ^87^Sr/^86^Sr ratios obtained by LA-ICP-MS on the Madagascar apatite, with laser ablation beam sizes ranging between 50 μm and 10 μm. (a) *Method 1* and (b) *Method 2* for data reduction. See caption of Fig. 5 for details. Solid and dashed red lines are the published mean reference value and associated 2 s.d., respectively, for the Madagascar apatite.^29^

Eighty-four analyses Madagascar apatite analyses are summarised in Fig. 6. The 2 s.e. of the ^87^Sr/^86^Sr ratio ranges between 120 ppm (50 μm) and 500 ppm (10 μm) for *Method 1*, and between 120 ppm (50 μm) and 890 ppm (10 μm) for *Method 2*. Repeated measurements with *Method 2* give average ^87^Sr/^86^Sr ratios that are all within error of both the reference value and our new solution value (50 μm: 0.711821 ± 0.000063; 25 μm: 0.711816 ± 0.000115; 13 μm: 0.711783 ± 0.000429; 10 μm: 0.711633 ± 0.000603; 2 s.d.) (Tables 1 and 2; ESI Table 2†). When *Method 1* is used, a shift towards higher ^87^Sr/^86^Sr is observed at 50 μm and 25 μm, whereas the average data at 13 μm and 10 μm are within error of the reference value (50 μm: 0.711957 ± 0.000060; 25 μm: 0.711963 ± 0.000092; 13 μm: 0.711930 ± 0.000214; 10 μm: 0.711827 ± 0.000344).

### 3.4. Slyudyanka

**Fig. 7:**
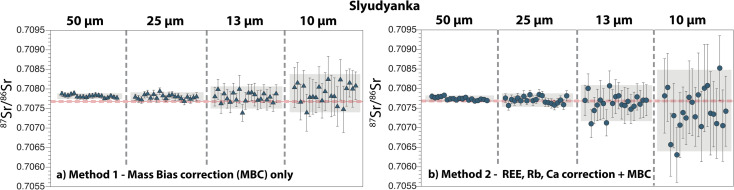
Compilation of ^87^Sr/^86^Sr ratios obtained by LA-ICP-MS on the Slyudyanka apatite, with laser ablation beam sizes ranging between 50 μm and 10 μm. (a) *Method 1* and (b) *Method 2* for data reduction. See caption of Fig. 5 for details. Solid and dashed red lines are the published mean reference value and associated 2 s.d., respectively, for the Slyudyanka apatite.^29^

Fig. 7 shows a summary of 85 Slyudyanka apatite analyses. The 2 s.e. of the ^87^Sr/^86^Sr ratio ranges between 100 ppm (50 μm) and 840 ppm (10 μm) for *Method 1*, and between 100 ppm (50 μm) and 1570 ppm (10 μm) for *Method 2*. Repeated measurements with *Method 2* give average ^87^Sr/^86^Sr ratios that are all within error of both the reference value and our new solution value (50 μm: 0.707743 ± 0.000082; 25 μm: 0.707706 ± 0.000173; 13 μm: 0.707629 ± 0.000460; 10 μm: 0.707439 ± 0.001042; 2 s.d.) (Tables 1 and 2; ESI Table 2†). When *Method 1* is used, a shift towards higher ^87^Sr/^86^Sr is observed at 50 μm and 25 μm, whereas the average data at 13 μm and 10 μm are within error of the reference value (50 μm: 0.707817 ± 0.000073; 25 μm: 0.707797 ± 0.000118; 13 μm: 0.707782 ± 0.000284; 10 μm: 0.707893 ± 0.000488).

### 3.5. Sumé

**Fig. 8:**
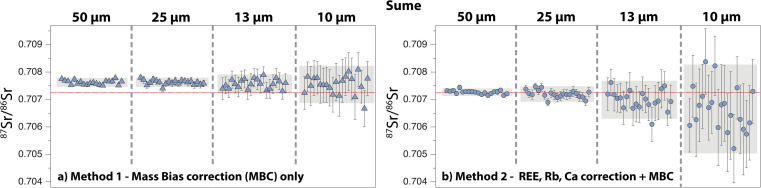
Compilation of ^87^Sr/^86^Sr ratios obtained by LA-ICP-MS on the Sumé apatite, with laser ablation beam sizes ranging between 50 μm and 10 μm. (a) *Method 1* and (b) *Method 2* for data reduction. See caption of Fig. 5 for details. The solid red line is the solution MC-ICP-MS value obtained in this study.

A summary of 83 Sumé apatite analyses is shown in Fig. 8. The 2 s.e. of the ^87^Sr/^86^Sr ratio ranges between 115 ppm (50 μm) and 1160 ppm (10 μm) for *Method 1*, and between 125 ppm (50 μm) and 2360 ppm (10 μm) for *Method 2*. Repeated measurements with *Method 2* give average ^87^Sr/^86^Sr ratios that are all within error of our new solution value but lower than the value of 0.7080 ± 0.0002 published by Lana *et al.*^77^ (50 μm: 0.707266 ± 0.000139; 25 μm: 0.707199 ± 0.000253; 13 μm: 0.706987 ± 0.000636; 10 μm: 0.706634 ± 0.001743; 2 s.d.) (Tables 1 and 2; ESI Table 2†). When *Method 1* is used, a shift towards higher ^87^Sr/^86^Sr is observed at 50 μm and 25 μm, whereas the average data at 13 μm and 10 μm are within error of the reference value (50 μm: 0.707624 ± 0.000163; 25 μm: 0.707394 ± 0.000170; 13 μm: 0.707274 ± 0.000422; 10 μm: 0.707303 ± 0.000752).

### 3.6. Ipirá

**Fig. 9:**
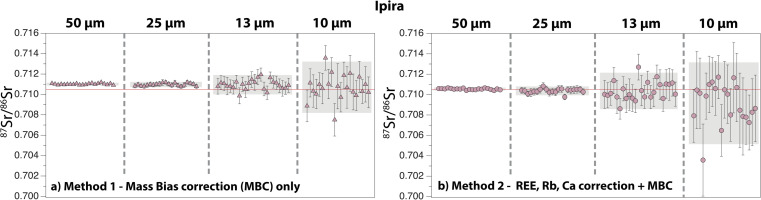
Compilation of ^87^Sr/^86^Sr ratios obtained by LA-ICP-MS on the Ipirá apatite, with laser ablation beam sizes ranging between 50 μm and 10 μm. (a) *Method 1* and (b) *Method 2* for data reduction. See caption of Fig. 5 for details. The solid red line is the solution MC-ICP-MS value obtained in this study.

Eighty-seven analyses were performed on this apatite standard, and the results are summarised in Fig. 9. The 2 s.e. of the ^87^Sr/^86^Sr ratio ranges between 155 ppm (50 μm) and 3115 ppm (10 μm) for *Method 1*, and between 190 ppm (50 μm) and 5340 ppm (10 μm) for *Method 2*. Repeated measurements with *Method 2* give average ^87^Sr/^86^Sr ratios that are all within error of our new solution value (50 μm: 0.710542 ± 0.000147; 25 μm: 0.710370 ± 0.000435; 13 μm: 0.710359 ± 0.001786; 10 μm: 0.709147 ± 0.004009; 2 s.d.) (Tables 1 and 2; ESI Table 2†). When *Method 1* is used, a shift towards higher ^87^Sr/^86^Sr is observed at 50 μm and 25 μm, whereas average data at 13 μm and 10 μm are within error of the reference value (50 μm: 0.711012 ± 0.000139; 25 μm: 0.710973 ± 0.000262; 13 μm: 0.710958 ± 0.000949; 10 μm: 0.710704 ± 0.002513).

### 3.7. Summary

Our new LA-MC-ICP-MS data for the Durango, Madagascar and Slyudyanka apatites are in good agreement with both our new solution values and published values for these standards (Fig. 5, 6 and 7; Tables 1 and 2). For Sumé, the ^87^Sr/^86^Sr obtained with both *Method 1* and *Method 2* are clearly lower than the value published by Lana *et al.*^77^ Importantly, these authors have noted that the Sumé apatite may not have a homogeneous ^87^Sr/^86^Sr depending on which sample batches are analysed. This is confirmed by our data, and the good agreement between our solution and laser ablation data for fragments issued from the same sample batch validates the robustness of our analytical approach. However, the presence of small Sr isotope heterogeneities in the five apatites analysed should not be ruled out at the micrometre scale, since a few individual analyses at 13 μm and 10 μm do not overlap reference values (Fig. 5, 6, 7, 8 and 9). Overall, the Durango, Madagascar, Slyudyanka, Sumé and Ipirá apatites, which have a large range of Sr and REE compositions as well as homogeneous Sr isotope ratios at the scale of the fragments analysed, constitute excellent reference materials for LA-MC-ICP-MS studies.

## 4. Discussion

### 4.1. Precision on the ^87^Sr/^86^Sr ratio

**Fig. 10:**
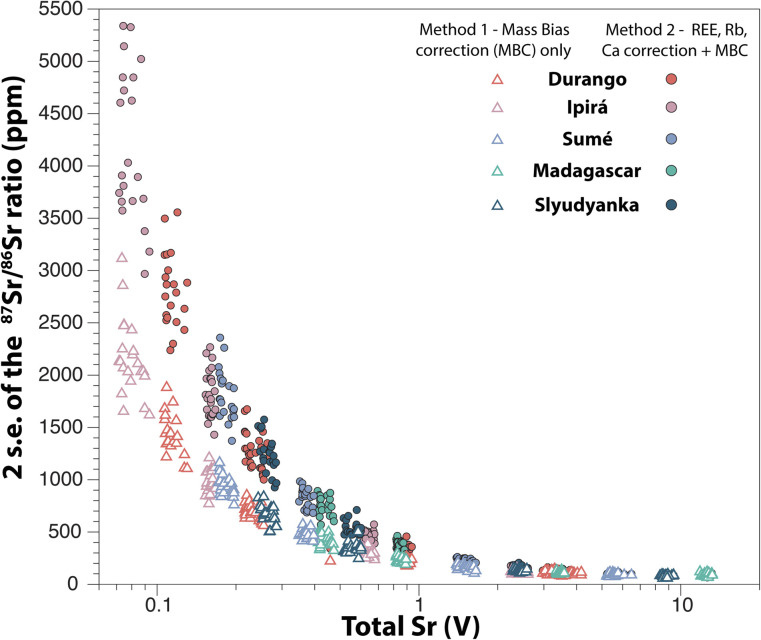
Precision on the ^87^Sr/^86^Sr ratio (given by the 2 s.e. of individual measurements) as a function of the total Sr intensity and the method used for data reduction.

The precision on the ^87^Sr/^86^Sr ratio critically depends on the total intensity measured on Sr, as summarised in Fig. 10. An internal precision better than 100 ppm (2 s.e.) is typically achieved for total Sr intensities greater than 8 V, and overall *Method 1* provides more precise data than *Method 2*. The better precision of *Method 1* is explained by the reduced number of data reduction steps compared to *Method 2*, and by the large uncertainty in the measurement of small intensities on half-masses (Fig. 3b and 4).

### 4.2. Accuracy and reproducibility of ^87^Sr/^86^Sr

**Fig. 11:**
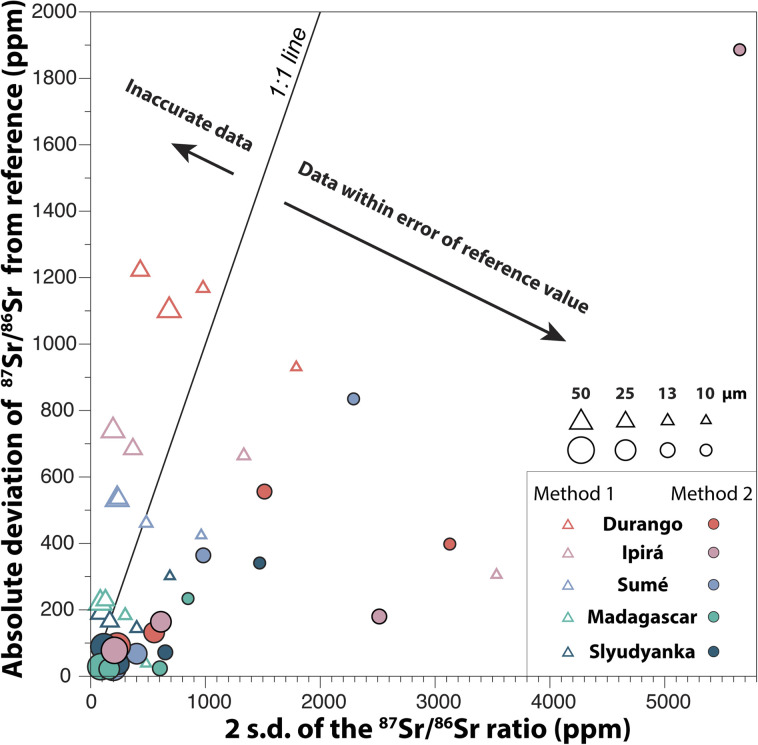
Changes in the absolute average deviation from the reference ^87^Sr/^86^Sr and in the 2 s.d. of the ^87^Sr/^86^Sr ratio, depending on which apatites are analysed, the size of the laser ablation beam and the method used for data reduction.

The average deviation from the reference ^87^Sr/^86^Sr of the five apatite standards is plotted in Fig. 11 as a function of the external reproducibility (given by the 2 s.d.) of the ^87^Sr/^86^Sr ratios measured at different laser ablation beam sizes. At 50 μm and 25 μm, both methods give similar external reproducibility, and the best 2 s.d. of *c.a.* 100 ppm is obtained in low REE/Sr apatites (*i.e.* Madagascar and Syudyanka). At smaller laser ablation beam sizes (*i.e.* 13 μm and 10 μm), a better reproducibility of the measurements is achieved with *Method 1*.

The 1 : 1 line in Fig. 11 separates the data that should be considered within error of the reference value, from data that should not. When *Method 1* is used for data reduction, the accuracy of the measurements is clearly affected for apatites with high REE/Sr ratios, such as Durango, Sumé and Ipirá. Analyses with 50 μm and 25 μm laser ablation beam sizes, which are the most precise in this study (Fig. 10), are significantly shifted from the reference value, by +190 to +1100 ppm (Fig. 11). When *Method 2* is used, the accuracy is greatly improved at 50 μm and 25 μm, although a substantial deviation from the reference value (of up to 2000 ppm) is observed for smaller ablation beams (*i.e.* 13 μm and 10 μm).

### 4.3. Importance of the limit of detection (LOD)

**Fig. 12:**
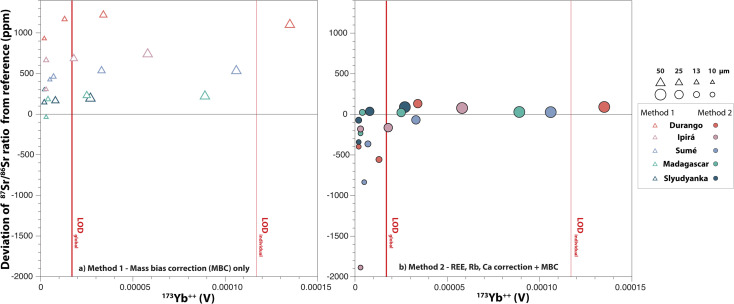
Average deviation from the reference ^87^Sr/^86^Sr ratio as a function of the ^173^Yb^++^ intensity measured on *m*/*z* 86.5. (a) *Method 1* and (b) *Method 2* for data reduction. Symbols and colour codes are same as in Fig. 11. The LOD_individual_ (thin red lines) is the average 3 s.d. of individual gas blank intensities, and the LOD_global_ (thick red lines) is the 3 s.d. of 460 gas blank analyses.

The average deviation from the reference ^87^Sr/^86^Sr of the five apatite standards is plotted as a function of the ^173^Yb^++^ intensity measured on *m*/*z* 86.5 in Fig. 12. The LOD_individual_ and LOD_global_, which were previously introduced in Section 3.1. (see Fig. 4), are reported in this figure, and it is striking that all analyses at 10 μm and 13 μm plot well below both the LOD_individual_ and LOD_global_. At 25 μm, 4 out of 5 apatite standards plot between the lower and upper LOD bounds (*i.e.* LOD_global_ and LOD_individual_, respectively) with only Slyudyanka plotting below the LOD_global_. At 50 μm, all apatites plot above the LOD_global_, with only Durango plotting above the LOD_individual._

The information combined from Fig. 11 and 12 indicate that when interfering species are measured below the LOD_global_, *Method 1* produces ^87^Sr/^86^Sr ratios with an acceptable level of accuracy. When interfering species are measured near or above the LOD_global_, *Method 2* delivers Sr isotope data with an excellent level of accuracy.

### 4.4. Comparison with previous studies

**Fig. 13:**
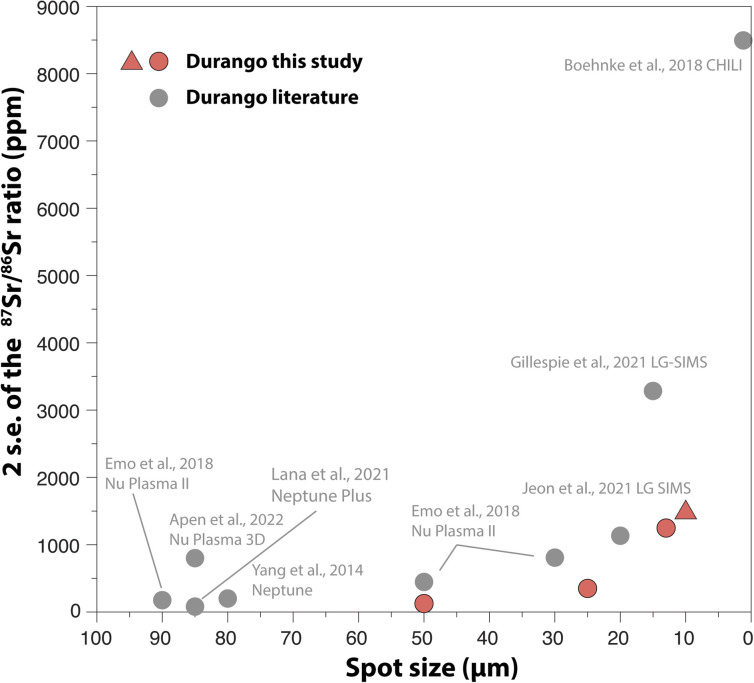
Precision on the ^87^Sr/^86^Sr ratio measured in Durango apatite with different mass spectrometry techniques. Red symbols: this study (triangles: *Method 1*; circles: *Method 2* for data reduction), and grey circles: literature data.^24,25,29,67,77,85,86^

A number of attempts have been made to measure ^87^Sr/^86^Sr ratios in apatite with a high spatial resolution, as summarised in Fig. 13. Overall, our approach provides a better precision for beam sizes between 50 μm and 10 μm, although a direct comparison with other studies would require a precise estimate of the volume of sample analysed. For instance, the volumes of apatite ablated in this study at 50 μm, 25 μm, 13 μm and 10 μm were around 75 000 μm^3^, 19 000 μm^3^, 5000 μm^3^, and 3000 μm^3^, respectively.

Amongst other techniques for high-precision analysis of Sr isotopes, the less destructive nature of the SIMS (Secondary Ion Mass Spectrometry) and CHILI (Chicago instrument for Laser Ionisation;^84^) techniques may be preferred for applications that require analyses of infinitesimal volumes of samples (*e.g.*, <100 μm^3^), such as in cosmochemistry. Our new data however demonstrate that LA-MC-ICP-MS platforms that are available in many laboratories worldwide can compete with other *in situ* techniques for Sr isotope measurements through faster acquisition and higher internal precision at comparable spatial resolution.

## 5. Conclusions and perspectives

The approach developed here allows precise and accurate ^87^Sr/^86^Sr ratios to be obtained when small volumes of apatite are ablated (*i.e.* 3000 to 75 000 μm^3^). Overall, both *Method 1* and *Method 2* for (LA-)MC-ICP-MS data reduction produce ^87^Sr/^86^Sr ratios with good precision and reproducibility. The accuracy of the ^87^Sr/^86^Sr strongly depends on both the composition of the ablated material and the method used for data reduction. As a guidance for method selection, three important points should be considered:

(i) *Method 1* can provide accurate and highly precise Sr isotope data when ^173^Yb^++^ intensity is measured below the LOD_global_.

(ii) *Method 2* greatly improves data accuracy when ^173^Yb^++^ intensity is measured near or above the LOD_global_. However, the uncertainty of individual measurements can be up to two times higher than with *Method 1*.

(iii) Analyses for which ^173^Yb^++^ intensity is close to the LOD_global_ should be treated with caution, and *Method 2* is recommended for more accurate (though less precise) results.

Overall, this analytical protocol for LA-MC-ICP-MS analyses and data reduction can be transposed to a wide range of applications that require a high spatial resolution, including the analysis of minute mineral inclusions, growth zones in rock-forming minerals, and growth zones in carbonates (*e.g.*, shells) and bio-apatite materials (*e.g.*, fish-otoliths, bones, teeth enamel).

## Author contributions

A. B. and B. D. designed the study and wrote the first draft of the paper. A. B., H. M. and B. D. performed LA-MC-ICP-MS analyses, and A. B. build data reduction schemes with help of B. D, H. M. and O. B.; D. B. and H. M. did the wet chemistry and O. B. and D. B. performed solution MC-ICP-MS analyses. All authors contributed to writing the final version of the paper.

## Conflicts of interest

There are no conflicts to declare.

## Supplementary Material

JA-038-D3JA00177F-s001

JA-038-D3JA00177F-s002

JA-038-D3JA00177F-s003
