# Supplementary material for: High spatial resolution (10–50 μm) analysis of Sr isotopes in rock-forming apatite by LA-MC-ICP-MS
Source: J Anal At Spectrom. 2023 Aug 29;38(10):2113–26. doi: 10.1039/d3ja00177f (PMC10549232; doi:10.1039/d3ja00177f)
Supplement: JA-038-D3JA00177F-s001 [file JA-038-D3JA00177F-s001.pdf]

Electronic Supplementary Material (ESI) for Journal of Analytical Atomic Spectrometry.
This journal is © The Royal Society of Chemistry 2023

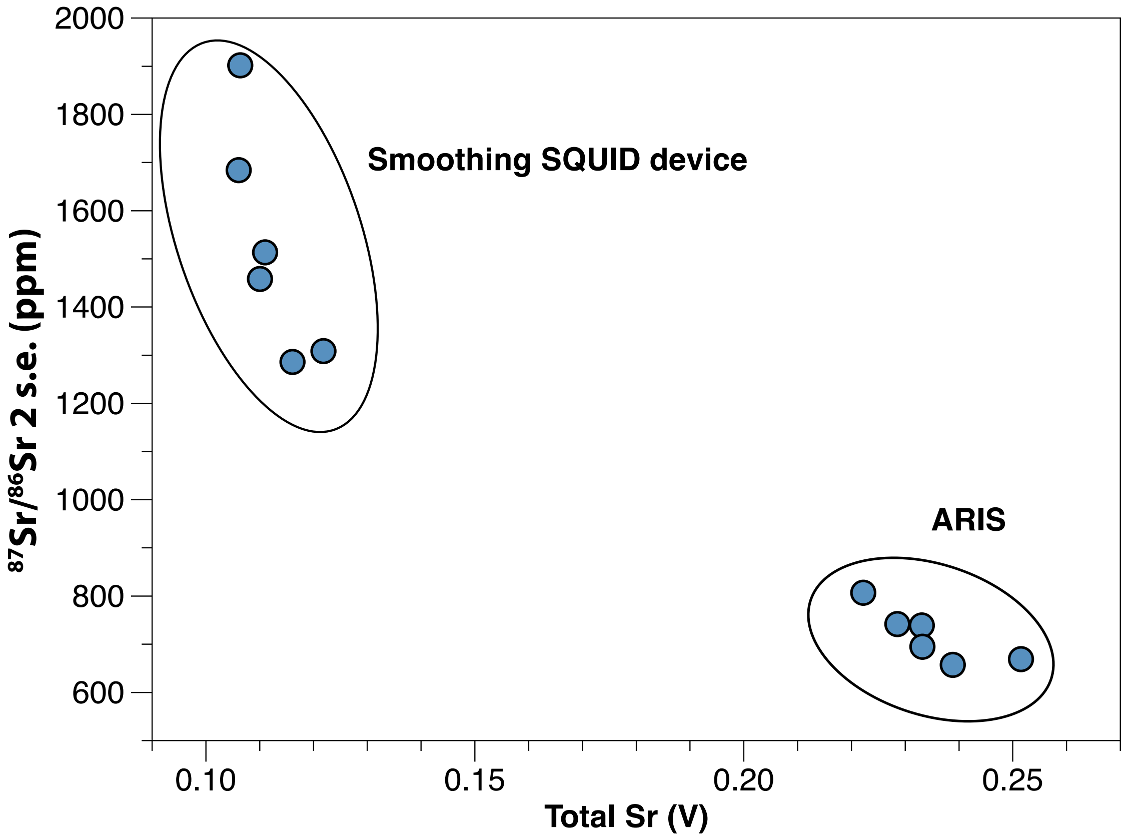

Supplementary Figure 1. Total Sr intensity and internal precision of Durango apatite analyses by LA-MC-ICP-MS with a 13x13 µm laser ablation beam, depending on the device used to transport the ablated material from the ablation cell to the plasma (i.e. smoothing SQUID or ARIS).
